# An analysis of the very high level of maternal distress experienced by South Korean women with young children

**DOI:** 10.1371/journal.pone.0274016

**Published:** 2022-09-21

**Authors:** Ji Yun Lee, Sae Eun Park, Yu-Mi Kim, Hong-Jun Cho, Young-Ho Khang

**Affiliations:** 1 The Support Team for the Early Life Health Management Project of Korea, Seoul, Korea; 2 The Support Team for the Seoul Healthy First Step Project, Seoul, Korea; 3 College of Nursing, Kangwon National University, Chuncheon, Korea; 4 Institute of Health Policy and Management, Seoul National University Medical Research Center, Seoul, Korea; 5 Department of Preventive Medicine, Hanyang University College of Medicine, Seoul, Korea; 6 Department of Family Medicine, Asan Medical Center, University of Ulsan College of Medicine, Seoul, Korea; 7 Department of Health Policy and Management, Seoul National University College of Medicine, Seoul, Korea; Flinders University, AUSTRALIA

## Abstract

This study was conducted as a part of a larger study to identify the needs of a maternal and early childhood home visit program that the South Korean central government has begun to expand nationwide. This survey measured the distress of mothers with children aged 2 years or younger during the transition into motherhood using the Being a Mother scale (BaM-13) and compared the stress levels for each question with those of existing studies in other countries. The survey results revealed that the mean BaM-13 score of the 350 participants was 17.09 (SD = 6.81), with 87.7% showing very high levels of maternal distress, indicated by a score of 9 or above in BaM-13. The item from the BaM-13 with the highest response rate of 2 or 3 points (sum of the percentage of those who answered 2 and 3 points) was “I have missed the life I had before I became pregnant with this baby/toddler,” to which 80.8% of the respondents agreed. The percentage of South Korean mothers who answered 2 or 3 points was higher for every item on the BaM-13 than that of Australian mothers. A comparison of the total BaM-13 score and 3 factors (child experience, adult’s experience, and emotional closeness) of the BaM-13 according to the participants’ characteristics revealed that discrepancies in women’s sociodemographic factors (including smoking and alcohol consumption behaviors) were not significant, whereas differences in the total BaM-13 score and the 3 factors of the BaM-13 according to the mothers’ scores on the Edinburgh Postnatal Depression Scale were noticeable. The high level of maternal distress observed in this study should be reflected in the nurse-led maternal and early childhood home visit program that the South Korean central government is expanding across the country.

## Introduction

Childbirth and childcare lead to considerable changes in the lives of many women. Childbirth is a major event that affects women both physically and psychologically. The transition to motherhood after childbirth is accompanied by significant changes in women’s health condition, quality of life, social relationships, and quality of social participation. At the same time, women often experience great stress during this period due to having to learn and perform their roles and functions as mothers [1].

Maternal distress or parenting stress refers to distress caused by the experience of pregnancy and childbirth, and is a specific type of stress felt when fulfilling one’s parental role due to hardships and demands experienced by mothers and parents in general, related to childcare [1–3]. Multiple studies on maternal distress in women have viewed it as a psychological and mental health problem and have measured it using the concepts of anxiety, depression, and adverse effects with a biomedical focus [1]. The level of maternal distress experienced by women varies depending on their socio-demographic characteristics, including economic status and educational level, and their personal resources and capabilities [1, 3–6]. It is also affected by family support and spousal relationships [1, 3–5]. It is, therefore, important to reduce the stress level of new mothers by managing stressors related to infant care.

Recent studies on maternal distress have focused on specific hardships that women experience during the transition into motherhood [1, 7]. These studies begin from the point of view that a woman’s experience of early parenthood should be understood as a normal response to a stressful situation that leads to major physical, social, and emotional changes [8, 9]. Rather than treating maternal distress as a mental or psychological problem that requires medical treatment or a resolution, or viewing it as an issue only experienced by vulnerable women who lack resources or capabilities, maternal distress should be understood as a universal experience women face. Thus, it is important to understand the attributes and contributing factors of maternal distress to help women transition into motherhood [1].

Many developed countries have implemented nurse-led maternal and early childhood home visit programs to assist women in the transition into motherhood and support children’s health and development [10–12]. Some examples of nurse-led maternal and early childhood home visit programs include the Nurse-Family Partnership program developed in the US [13] and the Maternal Early Childhood Sustained Home-Visiting (MECSH) program in Australia [14]. These programs utilize nurses as the main health care providers and offer intensive home-visiting services during the prenatal period until the child reaches the age of 2 years in order to support the health and development of children and the health and well-being of mothers, with the aim of strengthening families’ parenting competency. Australia’s MECSH program assesses not only children’s growth and development, but also changes in parenting stress that women experience during the transition into motherhood to evaluate the effects of the intervention program [14, 15].

South Korea (hereafter referred to as Korea) has also launched a maternal and early childhood home visit program in Seoul in 2013 [16]. The central government of Korea recently announced the Inclusive Nation’s Child Policy in 2019 and has expanded a maternal and early childhood home visit program nationwide for mothers with children aged 2 years or younger beginning in 2020 [17]. It is necessary to understand the level of stress that mothers with young children experience, as well as the specific factors related to their stress, in order to offer an effective maternal and early childhood home visit program. It has been reported that Korean women experience greater parenting difficulties compared to women from other countries, and Korea is known to have relatively poor systems and policies to support childcare compared to other countries [18].

This study was based on a survey [19] conducted as part of a larger study to identify the needs related to a maternal and early childhood home visit program promoted by the Korean central government after the passage of the Inclusive Nation’s Child Policy [17]. The purpose of this study was to measure the maternal distress experienced by mothers of children aged 2 years or younger during the transition into motherhood using the Being a Mother scale (BaM-13) [7]. The specific research objectives were 1) to identify the level of stress experienced by the mothers of children aged 2 years or younger during the transition into motherhood using the BaM-13 and determine if the BaM-13 scores of mothers varied depending on their characteristics and 2) to measure the stress level for each item of the BaM-13 and compare it with the findings of existing studies from other countries (Australia) [7].

## Methods

### Study subjects

The participants in this cross-sectional study were the same as the participants in a survey conducted as part of the study of maternal and early childhood home-visit services commissioned by the Ministry of Health and Welfare of Korea [19]. In order to investigate the needs of a home-visit program for mothers of children aged 2 years or younger, a national sample was selected. The number of samples was allocated by province based on data on the number of births per province collected by the Statistics Korea in 2017. A total of 490,000 women (25–45 years old) out of the 1,310,000 potential subjects registered with Macromill Embrain, a consulting and research firm, were stratified by region, and 4,520 women were selected through stratified random sampling. Invitations to participate were emailed to the selected women asking if they were willing to participate in an online survey. Among those who expressed their willingness to participate, 804 women who were pregnant at the time or had at least 1 child aged 2 years or younger were selected as the survey participants. Women without any child aged 2 years or younger were excluded. Prior to the start of the study, the participants were informed about the purpose of the study and the research institution. They were also informed that individual survey response results and personal information would be protected and that there was no penalty for refusing to participate or ending one’s participation early. The online survey questionnaire was only sent to those who had indicated their intention to participate (804 women), and they were provided a small reward after the response was completed. Since the survey was based on panel data of the research firm which made it possible to check each participant’s contact information, multiple participation of participations was not possible. A total of 613 pregnant women and women with young children completed the questionnaire. Of them, we excluded 5% (N = 31) of the participants whose response times were considered too fast. Then, we also excluded the participants who had extremely low or high scores or who answered insincerely to the subjective questions (e.g., writing consecutive Korean alphabets or numbers) (N = 82). Among remaining 500 participants including pregnant women, 350 mothers with at least 1 child aged 2 years or younger were selected as the final sample for this study. Considering difficulties in recruiting mothers with young children aged 24 months or less nationwide and the limitation of survey budget, we considered this number of study subjects acceptable for our study purpose. The survey was conducted from August 26 to August 30, 2019. The Seoul National University Hospital Institutional Review Board (IRB No. 1908-115-1056) approved the study.

### Questionnaire

The questionnaires collected the participants’ socio-demographic characteristics and were used to measure their maternal distress and depression levels. Socio-demographic characteristics included age, current number of children, area of residence, education level, average annual household income (1 USD = 1,000 KRW), and current status of cigarette smoking and alcohol consumption. Maternal distress was measured using the BaM-13 [7]. The level of depression was measured using the Edinburgh Postnatal Depression Scale (EPDS) [20].

We translated and back-translated the BaM-13, which was developed to assess new mothers’ experiences during the transition into motherhood, into Korean and used it to assess maternal distress. In Korea, this translated version of the BaM-13 was previously used to evaluate the effects of mothers’ group on maternal distress [21]. The BaM-13 consists of 13 total items that cover 3 domains (named 3 ‘factors’), including the child’s experience, the adult’s experience, and emotional closeness. Specifically, items about mothers’ feelings of loneliness or isolation, confidence about looking after the baby/toddler, closeness to the baby/toddler, a lack of support, coping, and guilty feeling are included. The respondents respond based on how they felt for the previous 2 to 3 weeks during the transition into motherhood on a Likert scale that ranges from 0 points to 3 points. For example, item 1 is “I have felt confident about looking after my baby/toddler,” and a score of 0 points indicates “Yes, most or all of the time,” 1 point indicates “Yes, some of the time,” 2 points indicates “No, not very often,” and 3 points indicates “No, rarely or never.” The total possible scores for the 13 items range from 0 points to 39 points, and a higher score indicates a greater level of maternal distress. In the Australian study, 9 points and above were considered to indicate possible high levels of distress [7]. The Cronbach’s α was 0.79 in the study by Matthey [7], 0.89 in the study by Park and June [21], and 0.88 in this study.

The Korean edition of the EPDS [20] was used to measure the level of depression of the respondents. This tool consists of 10 questions in total, including 8 questions related to depression and 2 questions related to anxiety, based on the subjective experiences of the respondents. Respondents choose the answers that come closest to how the respondents have felt in the past 7 days using a Likert scale that ranges from 0 points to 3 points. Total possible scores range from 0 points to 30 points, and a higher total score indicates a higher depression level. Major depression is indicated by a depression score of 13 points or more, and minor depression is indicated by a score of 10 to 12 points [22]. The Cronbach’s α in the study by Han and colleagues [22] was 0.85, while it was 0.90 in this study.

Korean and English versions of the full questionnaires are provided as an online supplement in the prior study examining the needs of home-visit program [19].

### Statistical analysis

Since we conducted an online survey preventing study participants from omitting or not answering a question, missing information on BaM-13 and other covariates used in this study did not exist for 350 participants. Univariable analysis was conducted for the survey participants’ socio-demographic characteristics, maternal distress, and depression levels. The percentages of respondents’ answers for the 13 individual items of the BaM-13, as well as the sum of the percentages of those who answered either 2 or 3 points (i.e., the percentage of those who answered 2 points + the percentage of those who answered 3 points) for each item, were calculated and presented. Crude mean scores and standard deviations (SDs) of the total BaM-13 score and scores for each of the 3 factors of the BaM-13 (child experience, adult’s experience, emotional closeness) [7] according to the participants’ characteristics were also presented. The t-test and analysis of variance were performed to test the discrepancy in BaM-13 scores according to the participants’ characteristics, and the *p*-values were calculated and presented. The mother’s age and parity were assumed to be related to maternal distress, so the two variables were adjusted as confounders, and the least-square means of BaM-13 scores were calculated using analysis of covariance. To determine the differences in the least-square means of BaM-13 scores according to the ordinal variables (education level, income level, EPDS score), the *p*-values for the trend observed using regression analysis were calculated and presented. Data on the individual items of BaM-13 from this study were compared to BaM-13 data from an Australian study [7]. All statistical analyses were performed using SAS version 9.4 (SAS Institute, Cary, NC, USA).

## Results

Table 1 shows the characteristics of the 350 participants. The majority (83.7%) of the total participants were in their 30s, and 55.1% had 1 child. Since stratified sampling was conducted based on regional population data released by Statistics Korea in 2017, the regional distribution of participants in this study was similar to the regional distribution of the number of births in Korea. In total, 74.3% of the participants had a bachelor’s degree or higher, 14.6% earned an annual household income of lower than 30,000 USD, 16.3% were current smokers, and 21.4% consumed alcohol more than 2 times per week. Among the participants, 29.1% had a total score of 13 points or higher on the EPDS, while 14.6% had a total score of 10 to 12 points (Table 1).

**Table 1:** Characteristics of the survey participants: 350 Korean women with young children.

Characteristics	No. of subjects (%)	Characteristics	No. of subjects (%)
Mother’s age (years)		Annual household income (USD)	
20–29	40 (11.4)	Less than 20,000	17 (4.9)
30–39	293 (83.7)	20,000–29,999	34 (9.7)
40–49	17 (4.9)	30,000–49,999	123 (35.1)
No. of children		50,000–69,999	107 (30.6)
1	193 (55.1)	70,000–999,999	52 (14.9)
2	129 (36.9)	100,000 or over	17 (4.9)
3 or more	28 (8.0)	Cigarette smoking status	
Region		Yes	57 (16.3)
Seoul	64 (18.3)	No	293 (83.7)
Gyeonggi-do/Gangwon-do	120 (34.3)	Alcohol consumption status (2+ times per week)	
Daejeon/Chungcheong-do	41 (11.7)	Yes	75 (21.4)
Gwangju/Jeolla-do/Jeju-do	38 (10.9)	No	275 (78.6)
Daegu/Gyeongsangbuk-do	33 (9.4)	Edinburgh Postnatal Depression Scale	
Busan/Gyeongsangnam-do	54 (15.4)	Less than 10	197 (56.3)
Education level		10–12	51 (14.6)
High school or lower	27 (7.7)	13 or over	102 (29.1)
Associate’s degree	63 (18.0)		
Bachelor’s degree	215 (61.4)		
Graduate school or more	45 (12.9)		

1 USD = 1,000 KRW

**Fig 1:**
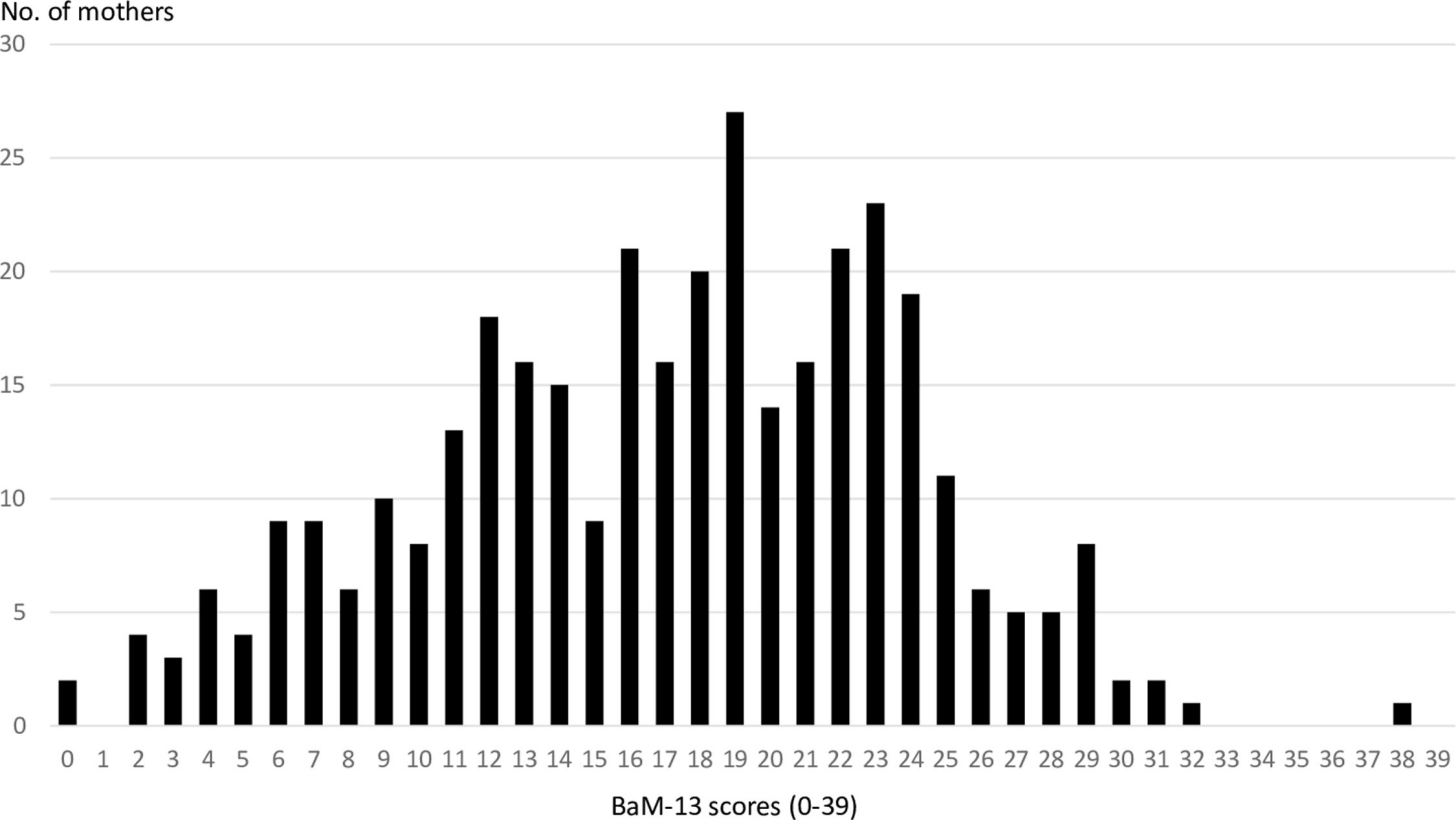
Distribution of BaM-13 scores* among 350 Korean women with young children. BaM-13, 13-item Being a Mother scale. *Higher scores indicate less satisfaction with the experience of motherhood.

Fig 1 shows the BaM-13 score distribution of the participants. The range of BaM-13 scores appeared as a bell curve, with a mode of 19 points. The median BaM-13 score among the survey participants was 18, and the mean score was 17.09 (SD = 6.81). In total, 87.7% of the participants received 9 points or more, and the proportion of those who scored twice as high (18 points) or above was 51.7%.

Table 2 lists the percentages of scores for each item, the percentages of those who gave scores of 2 or 3 points for each item, and the mean (SD) scores. The item for which the highest percentage of participants gave a score of 2 or 3 points was item 2 (“I have missed the life I had before I became pregnant with this baby/toddler”), with 80.8% of the total participants agreeing with the statement. The item with the next-highest proportion of participants citing agreement was item 12 (“I worry I am not as good as other mothers”), at 66.5%. This was followed by item 11 (“I have been annoyed or irritated with my baby/toddler”), at 57.4%., while 36.3% of participants agreed with the statement “I have felt lonely or isolated” (item 5) and 31.4% agreed with the statement “I have felt unsupported” (item 7). The question that corresponded to the lowest level of maternal distress was item 4 (“I have felt close to my baby/toddler”), with only 6.9% of participants answering negatively. Item 2 also had the highest mean score (2.07) of the individual BaM-13 items, and item 4 had the lowest mean score (0.59) (Table 2).

**Table 2:** Percentage (%) and mean score for each response* to the BaM-13 items among 350 Korean women with young children.

Item	% for each response option				% of 2 or 3 points	Mean scores (±SD)
	0	1	2	3		
1. I have felt confident about looking after my baby/toddler	15.7	54.3	25.4	4.6	30.0	1.19 (0.75)
2. I have missed the life I had before I became pregnant with this baby/toddler	3.7	15.4	51.4	29.4	80.8	2.07 (0.77)
3. I have found it hard to cope when my baby/toddler cries	13.4	37.4	44.9	4.3	49.2	1.40 (0.77)
4. I have felt close to my baby/toddler	48.9	44.3	6.3	0.6	6.9	0.59 (0.64)
5. I have felt lonely or isolated	26.6	37.1	32.3	4.0	36.3	1.14 (0.86)
6. I have felt bored	25.1	32.3	38.0	4.6	42.6	1.22 (0.88)
7. I have felt unsupported	28.6	40.0	25.7	5.7	31.4	1.09 (0.88)
8. I have felt alright about asking people for help or advice when I needed to	18.6	42.0	32.6	6.9	39.5	1.28 (0.84)
9. I have felt nervous or uneasy around my baby/toddler	18.3	34.6	42.3	4.9	47.2	1.34 (0.83)
10. I have been worried that something would happen to my baby	15.1	35.1	46.3	3.4	49.7	1.38 (0.78)
11. I have been annoyed or irritated with my baby/toddler	14.9	27.7	53.4	4.0	57.4	1.47 (0.79)
12. I worry I am not as good as other mothers	11.1	22.3	55.1	11.4	66.5	1.67 (0.82)
13. I have felt guilty	22.3	32.6	40.0	5.1	45.1	1.28 (0.87)
Total scores						17.09 (6.81)

BaM-13, 13-item Being a Mother scale; SD, standard deviation.

* Higher scores indicate less satisfaction with the experience of motherhood.

Table 3 shows the total BaM-13 scores according to the characteristics of the participants and the discrepancy in scores for the 3 subcategories of the BaM-13. The child’s experience is indicated by the sum of the scores for items 3, 9, 10, 11, 12, and 13. The adult’s experience is indicated by the sum of the scores for items 2, 5, 6, 7, and 8. Lastly, emotional closeness is indicated by the sum of the scores for items 1 and 4. The crude mean scores before adjusting for the age and parity of the participants and the least square mean scores after adjustment are shown. There was no meaningful difference in the total BaM-13 scores or the scores for the 3 factors before and after adjusting for the age and parity of the participants. As outlined in Table 3, there were no noticeable differences in the total BaM-13 scores and the scores for the 3 factors according to the participant’s characteristics tors (*p*>0.05 for all values). An increasing tendency in the total BaM-13 scores was observed as the income level decreased, but it was not statistically significant (*p* = 0.0534). A relatively high total BaM-13 score was observed in the province of Daegu/Gyeongsangbuk-do, but the discrepancy by region was not statistically significant (*p* = 0.2172). While mothers who smoked and drank alcohol tended to have relatively high BaM-13 scores, these differences were also not statistically significant (*p*>0.05 for all values). A statistically significant discrepancy in BaM-13 scores was observed according to the participants’ EPDS scores, with those who scored 13 points or higher on the EPDS having an average BaM-13 score approximately 9 points higher than that of the respondents who scored 10 points or less (22.67 versus 13.69 in the crude total BaM scores, respectively) (*p*<0.0001). This discrepancy in EPDS scores was found for all 3 factors of the BaM-13 (*p*<0.0001 for all values). Considering the multiple tests for the comparison of BaM scores (total score and scores by 3 factors) by study participants’ characteristics in Table 3, we may use the adjusted alpha level (0.05/4 = 0.0125) to consider the number of comparison for total BaM-13 scores and BaM scores for child experience, adult’s experience, and emotional closeness. However, when we applied this adjusted alpha level to the study findings, we could have similar conclusions.

**Table 3:** Crude and women’s age- and parity-adjusted least-square mean scores (95% confidence intervals) by three factors of BaM-13.

	Total BaM-13 score		Child experience		Adult’s experience		Emotional closeness	
	Crude (SD)	LS mean (SE)	Crude (SD)	LS mean (SE)	Crude (SD)	LS mean (SE)	Crude (SD)	LS mean (SE)
Mother’s age (years)								
20–34	17.12 (6.57)		8.54 (3.51)		6.87 (2.90)		1.71 (1.18)	
35–49	17.05 (7.14)		8.52 (3.73)		6.67 (3.09)		1.86 (1.20)	
*p*-value	0.9312		0.9732		0.5253		0.2304	
No. of children								
1	17.06 (6.76)		8.50 (3.59)		6.81 (2.92)		1.75 (1.19)	
2 or more	17.13 (6.88)		8.57 (3.62)		6.76 (3.06)		1.80 (1.20)	
*p*-value	0.9166		0.8449		0.8753		0.6889	
Region								
Seoul	16.17 (7.05)	16.10 (0.85)	8.03 (3.64)	8.01 (0.45)	6.48 (3.30)	6.45 (0.38)	1.66 (1.24)	1.63 (0.15)
Gyeonggi-do/Gangwon-do	17.38 (6.47)	17.30 (0.63)	8.53 (3.45)	8.52 (0.33)	6.91 (2.88)	6.88 (0.28)	1.93 (1.19)	1.91 (0.11)
Daejeon/Chungcheong-do	17.54 (7.22)	17.59 (1.07)	8.80 (4.28)	8.82 (0.56)	6.88 (2.67)	6.91 (0.47)	1.85 (1.04)	1.87 (0.19)
Gwangju/Jeolla-do/Jeju-do	16.89 (6.80)	17.08 (1.13)	8.58 (3.50)	8.63 (0.60)	6.68 (2.97)	6.76 (0.50)	1.63 (1.24)	1.69 (0.20)
Daegu/Gyeongsangbuk-do	19.42 (6.30)	19.52 (1.19)	9.94 (2.90)	9.97 (0.63)	7.48 (2.85)	7.52 (0.52)	2.00 (1.22)	2.03 (0.21)
Busan/Gyeongsangnam-do	15.93 (7.09)	15.94 (0.93)	8.02 (3.67)	8.02 (0.49)	6.44 (3.14)	6.45 (0.41)	1.46 (1.13)	1.47 (0.16)
*p*-value	0.2172	0.3349	0.1775	0.3223	0.6305	0.7672	0.1345	0.1409
Education level								
Associate’s degree or lower	18.27 (6.57)	18.33 (0.72)	9.06 (3.35)	9.06 (0.38)	7.30 (2.97)	7.34 (0.32)	1.91 (1.20)	1.93 (0.13)
Bachelor’s degree	16.80 (6.78)	16.81 (0.46)	8.40 (3.63)	8.41 (0.25)	6.66 (2.90)	6.66 (0.20)	1.74 (1.19)	1.74 (0.08)
Graduate school or more	16.16 (7.25)	15.95 (1.03)	8.11 (3.87)	8.07 (0.55)	6.38 (3.27)	6.29 (0.45)	1.67 (1.15)	1.60 (0.18)
*p*-value for trend	0.1393	0.2871	0.2459	0.5412	0.1396	0.2850	0.4191	0.2468
Annual household income (USD)								
Less than 30,000	18.45 (7.05)	18.68 (0.96)	9.12 (3.76)	9.18 (0.51)	7.43 (2.87)	7.53 (0.42)	1.90 (1.25)	1.97 (0.17)
30,000–49,999	17.64 (6.58)	17.63 (0.61)	8.88 (3.37)	8.87 (0.32)	6.96 (2.97)	6.96 (0.27)	1.80 (1.21)	1.80 (0.11)
50,000–69,999	16.96 (6.95)	16.95 (0.65)	8.41 (3.67)	8.41 (0.35)	6.75 (3.00)	6.74 (0.29)	1.80 (1.19)	1.80 (0.11)
70,000 or over	15.30 (6.56)	15.18 (0.82)	7.67 (3.66)	7.63 (0.44)	6.06 (2.94)	6.00 (0.36)	1.58 (1.10)	1.54 (0.14)
*p*-value for trend	0.0534	0.0969	0.0853	0.2050	0.0727	0.1271	0.4619	0.2400
Cigarette smoking status								
Yes	18.21 (6.22)	18.24 (0.91)	9.14 (3.50)	9.13 (0.48)	7.23 (2.65)	7.25 (0.40)	1.84 (1.16)	1.85 (0.16)
No	16.87 (6.90)	16.87 (0.40)	8.41 (3.61)	8.41 (0.21)	6.70 (3.03)	6.70 (0.17)	1.76 (1.20)	1.76 (0.07)
*p*-value	0.1752	0.4803	0.1629	0.5472	0.2208	0.5505	0.6385	0.3683
Alcohol consumption status (2+ times per week)								
Yes	17.97 (6.66)	18.01 (0.79)	9.19 (3.48)	9.19 (0.42)	6.99 (2.85)	7.01 (0.35)	1.80 (1.19)	1.81 (0.14)
No	16.85 (6.84)	16.84 (0.41)	8.35 (3.62)	8.35 (0.22)	6.73 (3.02)	6.73 (0.18)	1.77 (1.19)	1.76 (0.07)
*p*-value	0.2059	0.5149	0.0752	0.3341	0.5105	0.8028	0.833	0.3974
Edinburgh Postnatal Depression Scale								
Less than 10	13.69 (6.05)	13.66 (0.39)	6.99 (3.44)	6.98 (0.22)	5.32 (2.59)	5.31 (0.17)	1.38 (1.06)	1.37 (0.08)
10–12	19.10 (4.93)	19.14 (0.77)	9.45 (2.66)	9.46 (0.44)	7.75 (2.28)	7.76 (0.34)	1.90 (0.98)	1.92 (0.15)
13 or over	22.67 (4.57)	22.69 (0.55)	11.05 (2.60)	11.06 (0.31)	9.14 (2.19)	9.15 (0.24)	2.48 (1.18)	2.48 (0.11)
*p*-value for trend	< .0001	< .0001	< .0001	< .0001	< .0001	< .0001	< .0001	< .0001

BaM-13, 13-item Being a Mother scale; LS mean, least-square mean (age- and parity-adjusted mean); SD, standard deviation; SE, standard error.

**Fig 2:**
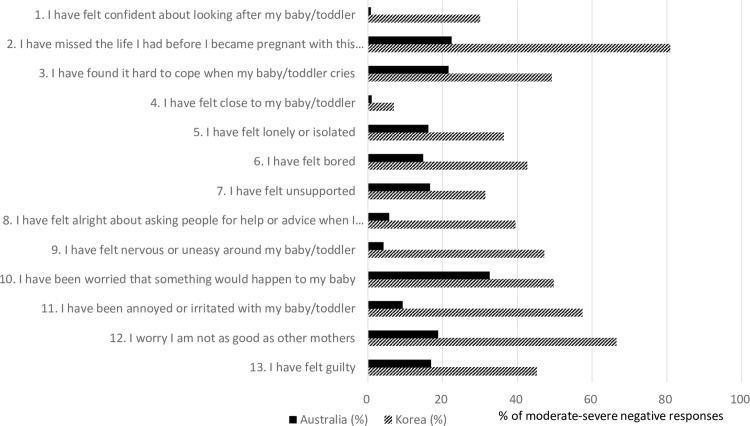
Comparison of each item from the BaM-13 between Korean and Australian mothers* based on the percentage of those who provided a moderate-to-severe negative response (2 or 3 points). *Australian data were from Matthey [7].

Fig 2 and the S1 Table compare the scores for the individual items of the BaM-13 from this study to those Australian mothers from a previous study by Matthey [7]. In this comparison, the percentages of Korean mothers who answered 2 or 3 points were higher for each individual BaM-13 item than those of Australian mothers. The biggest gap in percentage points (%p) between Australian and Korean mothers was observed for item 2 (“I have lost the life I enjoyed before I got pregnant”), with 80.8% of Korean mothers and only 22.4% of Australian mothers answering “Yes, most or all of the time” or “Yes, some of the time” (58.4%p). The next biggest gaps were observed for item 11 (“I have been annoyed or irritated with my baby/toddler;” 48.1%p), item 12 (“I worry I am not as good as other mothers;” 47.7%p), and item 9 (“I have felt nervous or uneasy around my baby/toddler;” 43.0%p) (Fig 2 and S1 Table). In addition, 30.0% of Korean mothers provided a negative answer for item 1 (“I have felt confident about looking after my baby/toddler”), while a significantly lower percentage (0.8%) of Australian women answered negatively.

## Discussion

The results of this study showed that the level of maternal distress experienced by Korean mothers of children aged 2 years or younger was very high. The mean total BaM-13 score in this study was 17.09 (SD = 6.81). The mean total BaM-13 score of 496 community mothers who visited Early Childhood Clinics in South West Sydney for routine baby-health checkups was 7.0 (SD = 5.4) [7], and in a study of 354 Saudi Arabian women who were recruited while waiting for their infant’s vaccination clinic appointment, it was 7.56 (SD = 6.03) [23]. The mean total BaM-13 score in this study was more than twice as high as the scores for women from those countries. The mean total BaM-13 score was 16.1 (SD = 6.4) in a study of women admitted to a mother and baby unit in Australia, which is similar to the results of this study [24]. The women in that study had been assessed as requiring parenting intervention by a physician and had received education and support related to raising an infant during a 5-day hospital stay [24]. The high BaM-13 score recorded in this study is unlikely to have been a coincidence since the BaM-13 score of the control group, which did not receive any intervention, was 15.50 (SD = 3.92) in a study conducted in Korea by Park and June [21] to evaluate the effectiveness of an intervention program utilizing mothers’ group.

It is unlikely that the high level of maternal distress found in this study was caused by problems related to the translated version of the BaM-13. Since the BaM-13, which was developed in Australia, was translated and back-translated into Korean, there is little chance that the 13 items in Korean differed from the English version. Multiple studies have observed that survey participants from Asia were more likely to provide midpoint responses on questionnaires [25, 26]. However, while responses for individual items in the BaM-13 ranged from 0 to 3 (3 = “Yes, most or all of the time,” 2 = “Yes, some of the time,” 1 = “No, not very often,” and 0 = “No, rarely or never”), the participants still had to choose between “Yes” and “No,” so there was no grey zone or midpoint response range. Therefore, the study’s findings might not be explained by the response patterns of Asian participants.

In addition, it is important to determine if the level of parenting stress of Koreans is high for other questionnaires in addition to the BaM-13. Using questionnaires other than the BaM-13, several studies have examined the differences in parenting stress between Korean parents and parents from other countries. A study on the differences in parenting stress (using the Parenting Stress Index-Short Form) and childhood problem behaviors between Korean and American mothers found that Korean mothers reported a significantly higher degree of parenting stress, yet a significantly lower degree of childhood problem behaviors, than American mothers [27]. One study examining the parenting stress (using the Parenting Stress Index-Short Form) and maternal-child interactions of married immigrant mothers from the Philippines, Vietnam, and Korea found that the level of parenting stress between Korean mothers in general and disadvantaged mothers from the Philippines and Vietnam living in Korea was similar [28]. In addition, an investigation conducted by the Korea Institute of Child Care and Education [18] surveyed 300 Korean households with children under 12 years old using the European Quality of Life Survey to measure parents’ quality of life to compare the quality of life of parents from Korea with parents from nations in the EU. The investigation concluded that the happiness level of Korean parents was lower than that of parents from 34 European countries, especially in terms of subjective wellbeing, standard of living, health, housing, work-life balance, education, childcare, social trust, and equality of household work. The results of existing studies corroborate the results of this study concerning Korean mothers’ high level of parenting stress.

There are several possible causes of the very high level of maternal distress experienced by Korean mothers. A recently coined term in Korea, “*dok-bak”* parenting, refers to a situation in which a mother takes care of her child or children by herself without any help from her husband, with very weak social support [29, 30]. In the past, the Confucian ideal of separation of the roles of men and women has contributed to an uneven parenting burden tilted toward mothers in Korea [31]. Until recently, depression has been considered a normal part of motherhood in Korea [31]. Although this Confucian ideal has faded in recent decades, Korea is known for having a low level of parental leave and virtually no paternity leave, relatively long working hours that limit fathers’ participation in parenting compared to other industrialized countries, and low levels of participation in parenting duties among fathers [18]. It was not until 2016 that items for governmental support for the diagnosis and treatment of antenatal and postnatal depression were officially included in the Maternal Child Health Act of Korea. Moreover, in the past, it was common for other family members such as grandmothers or grandmothers-in-law to assist new mothers caring for a newborn child; however, this type of familial support has eroded since the economic crisis in the late 1990s and subsequent disruption of familial bonds [32, 33]. Nuclear families have been the norm in Korea since the late 1990s. Fathers tend not to be very involved with childcare since there have been no large-scale proactive efforts to promote or mandate paternity leave. Although paternity leave is legally allowed, most employers do not support paternity leave, and women in the workforce usually try to return to their jobs after 3 months of maternity leave. While grandparents and in-laws usually play a supportive role for new mothers, changes in family structure often prevent mothers from taking advantage of the support of other family members. Changes concerning how women express their parenting stress also must be considered. In a society with a strong tradition of Confucianism that encourages an imbalanced relationship between men and women related to parenting, dissatisfaction among women has long been suppressed [31]. However, while gender equality has gained social acceptance in Korea, Korea has the world’s lowest national fertility rate [34], and the act of childbirth has been celebrated in recent years. Women now can strongly express and voice their dissatisfaction and distress in the process of becoming a mother and giving birth. Therefore, the factors contributing to the high level of maternal distress experienced by Korean mothers of children aged 2 years and younger reported in this study include (1) the lingering Confucian ideal regarding the role of women, (2) the erosion of familial parenting support due to changes in the family structure (from the extended family to the nuclear family), (3) the low level of paternity leave and subsequent low participation in parenting by husbands, (4) under-developed social support services such as home visiting services provided by nurses, and (5) Korea’s low fertility rate and associated changes in the cultural status of mothers and their expressions about hardships in motherhood.

Parenting stress among women is believed to be influenced by socio-demographic characteristics [35, 36]. According to a study by Kim and colleagues [37] on Korean women, the level of parenting stress experienced by mothers decreased as their level of income increased, and spousal participation in childcare was also inversely associated with women’s levels of parenting stress. Mothers who have completed a university degree or higher tend to experience less parenting stress than those with a high school diploma or lower [37]. Many studies, including a panel study on Korean children [35, 36], have observed that the level of household income and the mother’s education level was associated with parenting stress [38–40]. This is likely because the income and education level of mothers affect their access to resources and ability to cope with stress in emotionally difficult situations. However, some studies were unable to find any correlation between household income and parenting stress [6]. The analysis in this study revealed a discrepancy in the BaM-13 scores of mothers according to socio-demographic characteristics, though it was not statistically significant (*p*>0.05 for all values), which was partially due to the small sample size. The absolute differences in total BaM-13 scores according to socio-demographic variables were not considerably large. The difference between the highest annual household income level (70,000 USD and above) and the lowest annual household income level (less than 30,000 USD) was 3.15 points, and the difference in BaM-13 scores between the highest and lowest education level was approximately 2 points. This implies that the high level of parenting distress experienced by Korean women is prevalent in all social classes, and universal policies approaches are needed to alleviate parenting distress.

The characteristic that most strongly influenced the maternal distress in this study appeared to be depression, measured using the EPDS (*p*<0.0001). The discrepancy in the total BaM-13 score between those who received a score of 10 points or fewer on the EPDS (total BaM = 13.69) and those who received a score of 13 points or more (total BaM = 22.67) was 9 points. Our further analysis indicated that the correlation coefficient between EPDS score and BaM-13 score was 0.70 (*p*<0.0001). This suggests that the parenting stress of Korean mothers’ is closely related to depression. It also suggests that a maternal depression inventory can be useful for developing a more intensive maternal distress relief program and for assessing those in high-risk groups.

Parenting stress can be assessed using various tools, including the Parenting Stress Index developed by Abidin [41]. The BaM-13, which was used in this study, was created to assess the difficulties experienced by women during the transition into motherhood [7]. The responses for each item identify aspects related to childcare and emotional adjustment that women with young children experience. Based on the results of the survey, the percentage of those who answered 2 or 3 points was highest for item 2 (“I have missed the life I had before I became pregnant with this baby/toddler”), item 12 (“I worry I am not as good as other mothers”), and item 11 (“I have been annoyed or irritated with my baby/toddler”), in descending order. In addition, these were the items with the largest discrepancy in terms of the proportion of respondents who answered 2 or 3 points compared to the results of the same survey answered by Australian mothers [7]. A total of 80.8% of the participants in this study agreed with item 2, and this may be linked to social isolation after childbirth experienced by Korean women, the pressure of raising a child alone, and stress caused by a lack of personal time [42]. In particular, while women in modern Korean society, relative to the past, clearly recognize that childcare responsibilities should be shared between husband and wife, they are often struck by the reality that they are saddled with most of the responsibilities of childcare and are often frustrated by the unfairness of the situation and experience a psychological conflict. According to the survey results, the items that corresponded to the lowest level of childcare stress according to the percentage of respondents who answered 2 or 3 points were item 1 (“I have felt confident about looking after my baby/toddler”) and item 4 (“I have felt close to my baby/toddler”), both of which were from the “emotional closeness” subcategory of the 3 factors of the BaM-13. This suggests that Korean mothers feel relatively less hardships sharing emotional closeness with their children.

The responses to the individual items of the BaM-13 should be reflected in the intervention measures included in the maternal and early childhood home visit program that the Korean government is expanding nationwide. For example, the percentage of respondents who answered 2 or 3 points was considerably high for all items, and similar results were reported in a qualitative study on new Korean mothers with 12-month-old infants [42]. These results suggest that women are worried about the growth and development of their children and whether they are providing adequate childcare, and thus enhanced understanding of the child’s development. Therefore, addressing mothers’ negative experiences during their transition into motherhood may reduce their psychological and emotional stress. Given the above, the interventions included in the maternal and early childhood home visit program in Korea should include child health and development and approaches to address maternal distress related to childcare in nursing training, in order to build confidence in new mothers, improve their self efficacy, and provide adequate support for understanding and caring for an infant. Furthermore, the results of activities involved in interventions could be evaluated using the BaM-13.

This study has several limitations. First, this study compared the results of the BaM-13 between Korean women and Australian women [7]. This study analyzed the responses of mothers from the study sample with at least 1 child aged 2 years or younger who were recruited nationwide via email, whereas the Australian study examined 496 mothers from the community who visited Early Childhood Clinics in South West Sydney for routine infant-health checkups [7]. The method of the survey and the participants’ characteristics may have affected the results of the comparison between the women. Second, the size of the sample in this study was 350 mothers, which may have been too small to accurately identify discrepancies in total BaM-13 scores according to the full range of women’s characteristics. Third, the study participants were sampled from a research firm panel. We included those who indicated to their intention to participate and used data from participants whose information were considered reliable. Thus, the study participants may not be nationally representative. No additional methods for adjusting for non-representativeness of the sample were applied. Despite the limitation regarding sample representativeness, it should be noted that participants from all metropolitan cities and provinces in Korea were included through stratified random sampling reflecting the regional distribution of births in 2017.

## Conclusion

This study was conducted as part of a larger study that aimed to identify the needs of a maternal and early childhood home visit program. The level of distress that women experience during the transition into motherhood was assessed using the BaM-13 questionnaire. The results indicated that the level of parenting stress experienced by Korean mothers with young children was high across all social strata. The findings of studies like this should be reflected in the nurse-led home-visit program promoted by the Korean government.

## Supporting information

S1 Table Comparison of the 13-item Being a Mother scale between Korean and Australian mothers: Percentage of respondents who provided a moderate-to-severe negative response (2 or 3 points). (DOCX)
