# Supplementary material for: An analysis of the very high level of maternal distress experienced by South Korean women with young children
Source: PLoS One. 2022 Sep 21;17(9):e0274016. doi: 10.1371/journal.pone.0274016 (PMC9491576; doi:10.1371/journal.pone.0274016)
Supplement: S1 Table — (DOCX) [file pone.0274016.s001.docx]

**S1 Table. Comparison of the 13-item Being a Mother scale between Korean and Australian mothers**^*^**: percentage of respondents who provided a moderate-to-severe negative response (2 or 3 points)**

| Item | Korea (%) | Australia (%) | Difference (%p) |
| --- | --- | --- | --- |
| 1. I have felt confident about looking after my baby/toddler | 30.0 | 0.8 | 29.2 |
| 2. I have missed the life I had before I became pregnant with this baby/toddler | 80.8 | 22.4 | 58.4 |
| 3. I have found it hard to cope when my baby/toddler cries | 49.2 | 21.6 | 27.6 |
| 4. I have felt close to my baby/toddler | 6.9 | 1.0 | 5.9 |
| 5. I have felt lonely or isolated | 36.3 | 16.2 | 20.1 |
| 6. I have felt bored | 42.6 | 14.8 | 27.8 |
| 7. I have felt unsupported | 31.4 | 16.6 | 14.8 |
| 8. I have felt alright about asking people for help or advice when I needed to | 39.5 | 5.7 | 33.8 |
| 9. I have felt nervous or uneasy around my baby/toddler | 47.2 | 4.2 | 43.0 |
| 10. I have been worried that something would happen to my baby | 49.7 | 32.6 | 17.1 |
| 11. I have been annoyed or irritated with my baby/toddler | 57.4 | 9.3 | 48.1 |
| 12. I worry I am not as good as other mothers | 66.5 | 18.8 | 47.7 |
| 13. I have felt guilty | 45.1 | 16.9 | 28.2 |

^*^Australian data were from Matthey [1]

**Reference**

1. Matthey S. Assessing the experience of motherhood: The Being a Mother Scale (BaM-13). J Affect Disord. 2011;128;142-152.
